# Omitting radiotherapy in LUMINA-like breast cancer after breast conserving surgery: 10-year results of a population-based cohort study

**DOI:** 10.1093/oncolo/oyaf355

**Published:** 2025-10-22

**Authors:** Shenkangle Wang, Ziyu Zhu, Binbin Cui, Mingpeng Luo, Xixi Lin, Zijie Guo, Qingliang Wu, Linbo Wang, Xiaonan Sun, Jichun Zhou

**Affiliations:** Department of Surgical Oncology, Sir Run Run Shaw Hospital, Zhejiang University School of Medicine, Hangzhou, Zhejiang 310016, China; Biomedical Research Center and Key Laboratory of Biotherapy of Zhejiang Province, Hangzhou, Zhejiang 310016, China; Department of Radiation Oncology, Sir Run Run Shaw Hospital, Zhejiang University School of Medicine, Hangzhou, Zhejiang 310016, China; Department of Surgical Oncology, Sir Run Run Shaw Hospital, Zhejiang University School of Medicine, Hangzhou, Zhejiang 310016, China; Biomedical Research Center and Key Laboratory of Biotherapy of Zhejiang Province, Hangzhou, Zhejiang 310016, China; Department of Surgical Oncology, Taizhou Hospital of Zhejiang Province, Wenzhou Medical University, Linhai, Zhejiang Province, China; Department of Surgical Oncology, Sir Run Run Shaw Hospital, Zhejiang University School of Medicine, Hangzhou, Zhejiang 310016, China; Biomedical Research Center and Key Laboratory of Biotherapy of Zhejiang Province, Hangzhou, Zhejiang 310016, China; The First Affiliated Hospital of Zhejiang, Chinese Medical University, Hangzhou, Zhejiang 310014, China; Department of Surgical Oncology, Sir Run Run Shaw Hospital, Zhejiang University School of Medicine, Hangzhou, Zhejiang 310016, China; Biomedical Research Center and Key Laboratory of Biotherapy of Zhejiang Province, Hangzhou, Zhejiang 310016, China; Department of Surgical Oncology, Sir Run Run Shaw Hospital, Zhejiang University School of Medicine, Hangzhou, Zhejiang 310016, China; Biomedical Research Center and Key Laboratory of Biotherapy of Zhejiang Province, Hangzhou, Zhejiang 310016, China; Department of Surgical Oncology, Sir Run Run Shaw Hospital, Zhejiang University School of Medicine, Hangzhou, Zhejiang 310016, China; Biomedical Research Center and Key Laboratory of Biotherapy of Zhejiang Province, Hangzhou, Zhejiang 310016, China; Department of General Surgery, The Ninth People’s Hospital of Hangzhou, Hangzhou, Zhejiang 310014, China; Department of Surgical Oncology, Sir Run Run Shaw Hospital, Zhejiang University School of Medicine, Hangzhou, Zhejiang 310016, China; Biomedical Research Center and Key Laboratory of Biotherapy of Zhejiang Province, Hangzhou, Zhejiang 310016, China; Department of Radiation Oncology, Sir Run Run Shaw Hospital, Zhejiang University School of Medicine, Hangzhou, Zhejiang 310016, China; Department of Surgical Oncology, Sir Run Run Shaw Hospital, Zhejiang University School of Medicine, Hangzhou, Zhejiang 310016, China; Biomedical Research Center and Key Laboratory of Biotherapy of Zhejiang Province, Hangzhou, Zhejiang 310016, China

**Keywords:** breast cancer, LUMINA study, radiotherapy omission, breast-conserving surgery, population-based cohort study

## Abstract

**Background:**

Recently, the LUMINA trial reported 5-year results of omitting radiotherapy in a low-risk cohort after breast-conserving surgery (BCS). This study aimed to validate their 5-year results and investigate 10-year outcomes of patients satisfying their criteria in a population-based cohort.

**Materials and Methods:**

A total of 28 185 eligible patients were identified from the Surveillance, Epidemiology and End Results (SEER) database to establish SEER-LUMINA cohort. The Kaplan–Meier method was employed to estimate recurrence incidence and survival outcomes. Matched cohorts were generated using propensity score matching (PSM). The Cox proportional hazards model was used to generate hazard ratios.

**Results:**

A total of 6808 patients of the 28 185 did not receive radiotherapy while 21 377 underwent postoperative radiotherapy. After PSM, each group had 5954 patients, revealing significant differences in local recurrence incidence (no-radiotherapy group 0.65% at 5 years and 3.68% at 10 years vs radiotherapy group 0.14% at 5 years and 1.54% at 10 years, *P *< .001), breast cancer-specific survival (BCSS) (no-radiotherapy group 98.51% at 5 years and 96.11% at 10 years vs radiotherapy group 99.05% at 5 years and 96.34% at 10 years, *P *= .028). No difference was observed in BCSS between the 2 groups for SEER-LUMINA patients with tumor ≤1.4 cm (the optimal tumor size cutoff value for BCSS) (*P *= .099).

**Conclusion:**

Incidence, whether 5-year or 10-year recurrence, was lower than 5% in the SEER-LUMINA cohort. Radiotherapy following BCS reduced the local recurrence statistically significantly for those patients, but the clinical impact of this reduction was modest. Radiotherapy did not result in a difference in BCSS for SEER-LUMINA patients with tumors ≤1.4 cm.

Implications for Practice Our study evaluated long-term benefits of adjuvant radiotherapy for LUMINA-like patients and demonstrated the desirability of omitting radiotherapy in selected patients in the real world. This evidence supports the application of radiotherapy omission in LUMINA-like patients, especially those with tumors ≤1.4 cm, potentially reducing treatment-related side effects while maintaining similar survival outcomes.

## Introduction

Radiotherapy is an important part of treatment for early-stage breast cancer following breast-conserving surgery (BCS).^1^ Multiple studies have shown that postoperative radiotherapy can significantly improve the prognosis of breast cancer patients.^2–6^ However, radiotherapy can cause side effects such as breast induration, telangiectasia, and even ischemic heart disease.^7^^,^^8^ Besides, a meta-analysis indicated radiotherapy benefit extent is associated with patient age, tumor grade, estrogen receptor (ER) status, and endocrine treatment, with younger patients and high-grade tumor cases benefiting more.^9^ Therefore, it is necessary to precisely select the appropriate population for radiotherapy omission.

De-escalation therapies have gained attention in early-stage breast cancer treatment for their potential to minimize side effects while maintaining efficacy. Many studies have explored de-escalation in various aspects of breast cancer treatment. For example, the SOUND and the INSEMA trials confirmed the safety of omitting axillary surgery in low-risk patients.^10^^,^^11^ In terms of hormone therapy, the EUROPA trial aimed to compare radiotherapy and hormone therapy as monotherapies with regard to their impact on the prognosis of patients with luminal A breast cancer.^12^ Besides, several studies have focused on omitting radiotherapy in older patients with early-stage breast cancer after BCS.^13–17^ The Cancer and Leukemia Group B (CALGB) 9343 trial revealed that despite a higher incidence of local recurrence for patients aged ≥70, T1N0M0, ER-positive, omission of radiotherapy did not impact their overall survival (OS) or breast cancer-specific survival (BCSS).^14^ The National Comprehensive Cancer Network (NCCN) adopted this trial, stating that radiotherapy can be omitted in this population.^18^ Another pivotal study, the PRIME II trial, enrolled patients aged ≥65 with tumors ≤3 cm and hormone receptor-positive. These patients who had undergone BCS were randomly assigned to 2 groups: those with and those without radiotherapy. No significant difference in terms of BCSS was observed between the 2 groups. However, the 10-year cumulative incidence of local recurrence was 9.5% for the no-radiotherapy group, compared to only 0.9% for the radiotherapy group.^19^

Recently, the LUMINA trial found that the 5-year recurrence incidence was less than 5% for women aged ≥55 with T1N0, grade 1 or 2, luminal A breast cancer who had undergone BCS and received only endocrine treatment, even without adjuvant radiotherapy.^13^ However, this trial was a single-arm study and lacked comparison with patients who received radiotherapy. In addition, it has been pointed out that the results with only a 5-year follow-up are not sufficient to support exempting LUMINA patients from radiotherapy, as the recurrence may increase significantly after 5 years.^20^^,^^21^ Indeed, studies have shown that breast cancer after surgery has 2 recurrence peaks at 3 and 7 years,^22^ and ER-positive breast cancer has a higher risk of late recurrence.^23^ Thus, long-term follow-up results of LUMINA patients will provide more solid evidence for the safety of radiotherapy omission. Besides, the patients enrolled in this trial were highly selected, not fully representing the real-world population.^24^ LUMINA patients not undergoing radiotherapy were also expected to receive endocrine treatment, but studies have noted that oral medication adherence in clinical trial populations tends to be higher than in the real-world population.^25^^,^^26^ Thus, applying the results of the LUMINA trial to the general population requires caution. Fortunately, population-based studies can partially compensate for this shortcoming and provide external validity.^24^

The present study established a population-based cohort from the Surveillance, Epidemiology and End Results (SEER) database that potentially meets the LUMINA criteria (SEER-LUMINA cohort), assessing the impact of omitting postoperative radiotherapy on this population in the long term. By comparing SEER-LUMINA patients who received radiotherapy and those who did not, we further evaluated the benefits of adjuvant radiotherapy for them and demonstrated the desirability of omitting radiotherapy in LUMINA patients in the real world.

## Methods

### Patient selection and data sources

**Figure 1.**
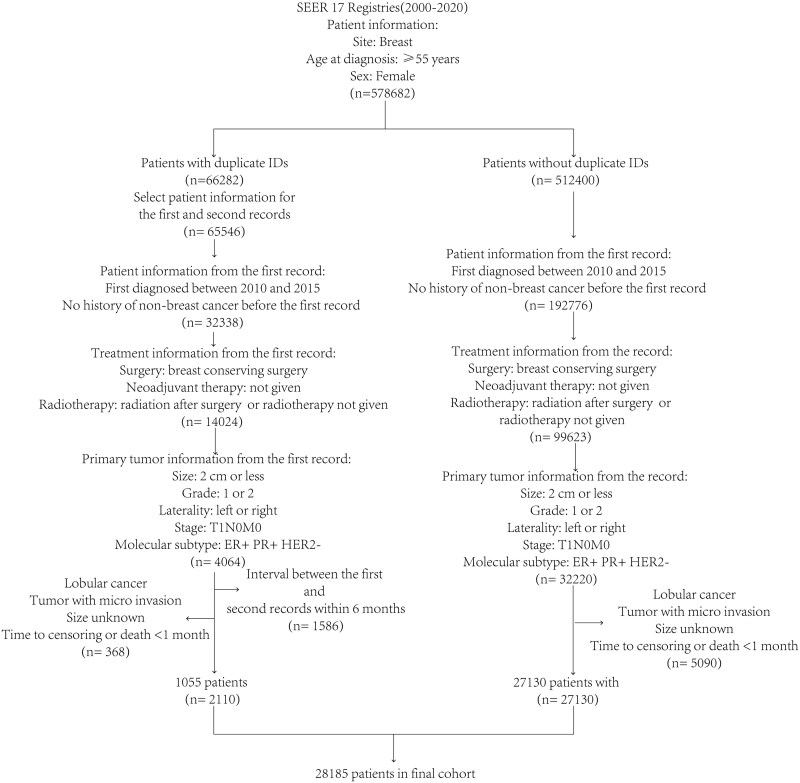
The flow diagram of participant inclusion and exclusion.

Cancer incidence data of female breast cancer patients diagnosed between 2010 and 2020 were collected from the SEER 17 registry database. Data were further extracted for the first record between 2010 and 2015 according to inclusion and exclusion criteria (Supplementary Material) (Figure 1). Eligible patients were female breast cancer patients after BCS who were ≥55 years and had T1N0M0, grade 1 or 2, ER-positive and PR-positive and HER2-negative tumor. Institutional review approval was not required because patients’ records from SEER database are anonymous.

### Patient outcomes

Local recurrence was defined as the recurrence of invasive cancer or DCIS in the ipsilateral breast according to LUMINA trial.^13^ Definition and censoring for contralateral breast cancer, BCSS, disease-free survival (DFS), and OS can be found in Supplementary Material. Information on recurrence were derived from SEER as previously described.^27–29^

This study evaluated differences in local recurrence risk by tumor grade (grade 1, grade 2), tumor size (≤1.0 cm, >1.0 cm), age at diagnosis (young: <65 years, old: ≥65 years), chemotherapy (no/unknown, yes), laterality (left, right), race (Asian or Pacific islander, Black, unknown/others, White), primary site (upper-outer, central portion and nipple, lower-inner, lower-outer, others, upper-inner), histologic cancer type (ductal, mucinous, other, tubular), and year of diagnosis (2010-2012 and 2013-2015) in the 2 group using log-rank tests. Furthermore, hazard ratios (HRs) were calculated for these factors as well as use of radiotherapy in the matched cohort.

### Sensitivity analysis

It has been reported that some low-risk patients in SEER died from breast cancer without experiencing recurrence, which could be due to their recurrence not being diagnosed or recorded.^30^ Because this could lead to an underestimation of the recurrence incidence, we assumed that these patients must have experienced recurrence during the period from breast cancer diagnosis to death from breast cancer. Kaplan–Meier analysis with interval censoring was used for calculations.

### Statistical analysis

The Kaplan–Meier method was used to estimate local recurrence, contralateral breast cancer, BCSS, and OS for patients who did not undergo radiotherapy. Pearson’s chi-square test was used to compare the distribution of variables across no-radiotherapy radiotherapy groups. For PSM, matched variables included age, year of diagnosis, chemotherapy, tumor size, tumor grade, histological type, site of primary cancer, laterality, and race. The Cox proportional hazards model was used to generate HR. The model’s proportional hazards assumption was assessed with the Schoenfeld residuals. An optimal cutoff value for tumor size based on BCSS was determined using the X-tile software.^31^ Pearson’s chi-square test was performed in SPSS 25.0 software. PSM was conducted using the “MatchIt” package in R software (version 4.2.1), with survival analysis using “survminer” and “survival” packages, and the “interval” package for interval-censored survival analysis.

### Role of the funding source

The funding source had no involvement in manuscript writing or in any other aspect related to the study.

## Results

### Characteristics of patients

Table 1 shows characteristics of the patients included in the study. Before PSM, differences were observed between the no-radiotherapy and radiotherapy groups in most variables. The median age in the no-radiotherapy group was higher than that in the radiotherapy group, with medians of 74 years (interquartile range [IQR] 67-81 years) for the no-radiotherapy group and 66 years (IQR 61-71 years) for radiotherapy group. The greatest distribution disparity was observed in group ≥80 years. Less than 4% of patients in both groups received chemotherapy, with a slightly lower percentage in the no-radiotherapy group. In accordance with the methods of a prior study,^32^ 92.1% patients were confirmed to have undergone sentinel lymph node biopsy (SLNB) alone. In the radiotherapy group, 91.8% of patients underwent SLNB, compared to 93.2% in the no-radiotherapy group. Additionally, the no-radiotherapy group had a higher proportion of patients with tumor size ≤0.5 cm and those with grade 1.

**Table 1.** Characteristics of the patients at baseline.

Variable	Received radiotherapy after breast conserving surgery?		*P*
	No (*n* = 6808)	Yes (*n* = 21 377)	
**Age, *n* (%)**			<.001
** 55 to <60 years**	534 (7.8)	4057 (19.0)	
** 60 to <65 years**	682 (10.0)	5197 (24.3)	
** 65 to <70 years**	908 (13.3)	5290 (24.7)	
** 70 to <75 years**	1326 (19.5)	3468 (16.2)	
** 75 to <80 years**	1250 (18.4)	2088 (9.8)	
** ≥80 years**	2108 (31.0)	1277 (6.0)	
**Year of diagnosis, *n* (%)**			<.001
** 2010**	864 (12.7)	3035 (14.2)	
** 2011**	1027 (15.1)	3506 (16.4)	
** 2012**	1136 (16.7)	3655 (17.1)	
** 2013**	1185 (17.4)	3745 (17.5)	
** 2014**	1260 (18.5)	3505 (16.4)	
** 2015**	1336 (19.6)	3931 (18.4)	
**Chemotherapy, *n* (%)**			<.001
**No/Unknown**	6680 (98.1)	20 544 (96.1)	
** Yes**	128 (1.9)	833 (3.9)	
**Tumor size, *n* (%)**			.103
** ≤0.5 cm**	1005 (14.8)	2938 (13.7)	
** 0.5-1.0 cm**	2669 (39.2)	8438 (39.5)	
** 1.1-2.0 cm**	3134 (46.0)	10 001 (46.8)	
**Tumor grade, *n* (%)**			<.001
**Well differentiated; Grade 1**	3641 (53.5)	10 798 (50.5)	
**Moderately differentiated; Grade 2**	3167 (46.5)	10 579 (49.5)	
**Histologic cancer type, *n* (%)**			<.001
** Ductal**	6081 (89.3)	19 871 (93.0)	
** Tubular**	118 (1.7)	312 (1.5)	
** Mucinous**	419 (6.2)	817 (3.8)	
** Other**	190 (2.8)	377 (1.8)	
**Primary site, *n* (%)**			<.001
** Upper-outer**	2312 (34.0)	7681 (35.9)	
** Upper-inner**	1039 (15.3)	3493 (16.3)	
** Lower-outer**	493 (7.2)	1585 (7.4)	
** Lower-inner**	478 (7.0)	1486 (7.0)	
**Central portion and nipple**	231 (3.4)	745 (3.5)	
** Other**	2255 (33.1)	6387 (29.9)	
**Laterality, *n* (%)**			.523
** Left**	3444 (50.6)	10 717 (50.1)	
** Right**	3364 (49.4)	10 660 (49.9)	
**Race, *n* (%)**			.017
** White**	5812 (85.4)	18 280 (85.5)	
** Black**	427 (6.3)	1246 (5.8)	
**Asian or Pacific Islander**	485 (7.1)	1658 (7.8)	
** Unknown/other**	84 (1.2)	193 (0.9)	

### Comparison of treatment outcomes for no-radiotherapy/radiotherapy patients before PSM

**Figure 2.**
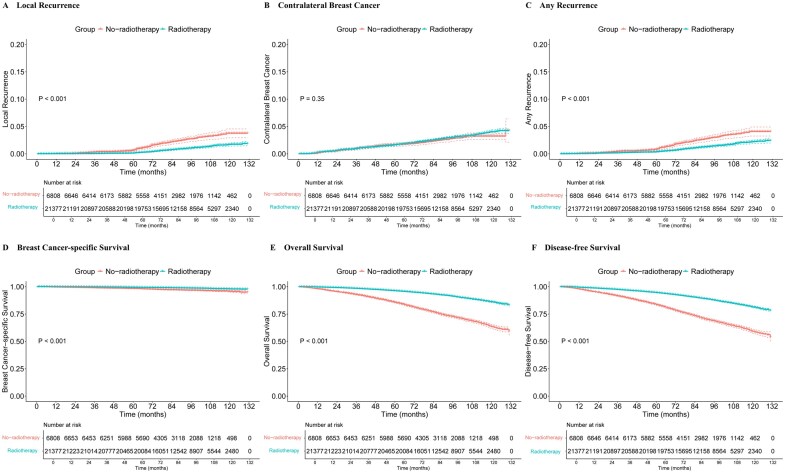
Cumulative incidence of (A) local recurrence, (B) cancer in the contralateral breast, (C) any recurrence, (D) BCSS, (E) OS, and (F) DFS before PSM. BCSS = breast cancer-specific survival, DFS = disease-free survival, OS = overall survival, PSM = propensity score matching.

The 5-year and 10-year cumulative incidence of local recurrence was 0.64% and 3.75% for no-radiotherapy patients and 0.12% and 1.68% for radiotherapy patients, respectively (*P* < .001) (Figure 2A) (Table S1). For 5-year local recurrence, the absolute risk reduction (ARR) in the radiotherapy group was 0.52%, with a number needed to treat (NNT) of 192 by method according to Laupacis et al.^33^ The ARR of 10-year local recurrence was 2.07%, and the NNT was 48. The 2 groups did not differ in contralateral breast cancer incidence (*P *= .350) (Figure 2B). No-radiotherapy group had significantly more any recurrence (*P *< .001) (Figure 2C). The 5-year and 10-year cumulative incidence were listed in Table S1. Radiotherapy patients also displayed significantly better BCSS (*P *< .001), OS (*P *< .001), and DFS (*P *< .001) than no-radiotherapy patients (Figure 2D-F) (Table S1).

### Comparison of treatment outcomes for no-radiotherapy/radiotherapy patients after PSM

To balance characteristics between the 2 groups, 1:1 PSM was used to generate a matched cohort (Table S2). Primary site (*P *= .014) and race (*P *= .047) were the only variables showing significant difference between the 2 groups after PSM. The median age after PSM was 73 years for the no-radiotherapy group and 72 years for the radiotherapy group.

**Figure 3.**
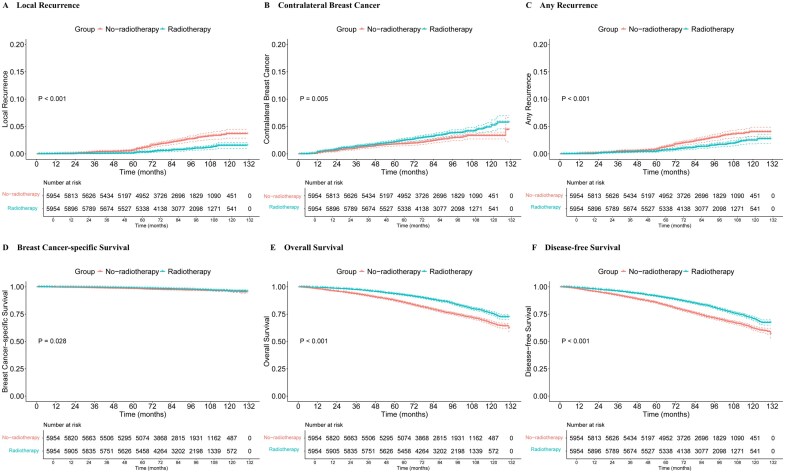
Cumulative incidence of (A) local recurrence, (B) cancer in the contralateral breast, (C) any recurrence, (D) BCSS, (E) OS, and (F) DFS after PSM. BCSS = breast cancer-specific survival, DFS = disease-free survival, OS = overall survival, PSM = propensity score matching.

After PSM, the 5-year and 10-year cumulative incidence of local recurrence were 0.65% and 3.68% for no-radiotherapy patients and 0.14% and 1.54% for radiotherapy patients, respectively (*P* < .001) (Figure 3A) (Table S1), with an ARR of 0.51% at 5 years and 2.14% at 10 years and an NNT of 196 at 5 years and 47 at 10 years. The 5-year and 10-year incidence of contralateral breast cancer were higher in the radiotherapy group, with a *P* value of .005 (Figure 3B). No-radiotherapy group had significantly more any recurrence (*P *< .001) (Figure 3C). The 5-year and 10-year cumulative incidence was listed in Table S1. There were also significant differences between the 2 groups in BCSS (*P *= .028), OS (*P *< .001), and DFS (*P *< .001) (Figure 3D-F) (Table S1).

### Risk of local recurrence

**Figure 4.**
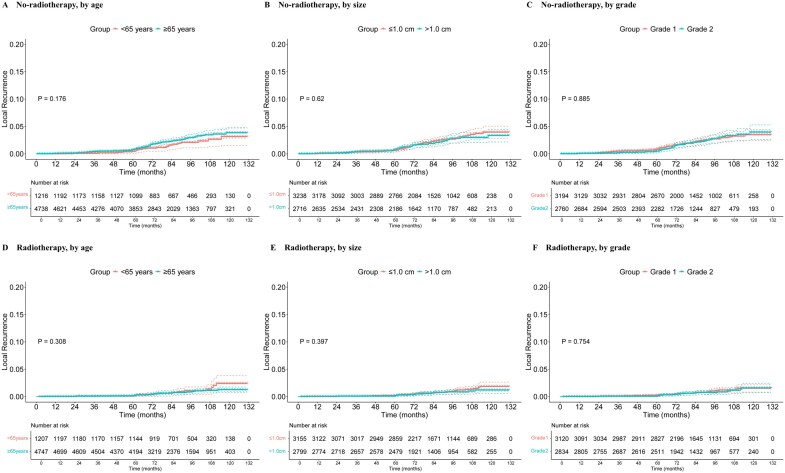
Risk of local recurrence in no-radiotherapy and radiotherapy patients. The cumulative risk of local recurrence in the SEER cohort is stratified by (A, D) age at diagnosis, (B, E) tumor size, and (C, F) tumor grade. SEER = Surveillance, Epidemiology and End Results.

Among no-radiotherapy patients, there was no statistical difference in local recurrence between those aged <65 years and those aged at least 65 years (*P *= .176) (Figure 4A). No significant differences were found in local recurrence risk by tumor size, tumor grade (Figure 4B-C), or other factors (Figure S1). Among radiotherapy patients, there was no statistical difference in local recurrence between the 2 age groups (*P *= .308) (Figure 4D). Similarly, no statistically significant differences were observed in local recurrence risk by other factors (Figure 4E-F, Figure S2).

Univariate Cox proportional hazards analyses were conducted based on potential risk factors for local recurrence, selecting variables with *P *< .1 for multivariate Cox proportional hazards analyses. Radiotherapy was the only prognostic factor for lower risk of local recurrence (radiotherapy vs no radiotherapy [HR 0.35, 95% CI 0.25-0.49, *P *< .001]) (Table S3). Tumor location in the lower-outer quadrant was of borderline significance for predicting local recurrence (lower-outer quadrant vs upper-outer quadrant [HR 1.70, 95% CI 0.99-2.92, *P *= .053]).

### Identification of a cohort that may not benefit from radiotherapy

Survival benefits provided by radiotherapy were statistically significant. We therefore sought to identify a subgroup from SEER-LUMINA patients at least 55 years of age that may not benefit from radiotherapy. BCSS instead of OS was used to assess patient survival outcomes because no-radiotherapy group displayed a significantly higher incidence of non-breast-cancer-related death (Figure S3) and SEER does not provide information on comorbidities.

A tumor >1.0 cm was identified as one of the risk factors of BCSS (tumor >1.0 cm vs tumor ≤1.0 cm [HR 1.75, 95% CI 1.36-2.24, *P* < .001]), suggesting that patients with small tumors were at low risk. In all included patients, 1.4 cm was identified as the optimal tumor size cutoff value for BCSS using X-tile software (Figure S4). PSM was used to generate a matched cohort consisting of patients with tumor ≤1.4 cm collected from all included patients (Table S4). No statistical difference in BCSS between radiotherapy and no-radiotherapy patients was observed in this cohort (*P *= .099) (Figure S5).

### Sensitivity analysis

It was assumed that patients who died of breast cancer without a record of recurrence must have experienced recurrence during the period from the diagnosis of breast cancer to death. If the recurrence concerned only ipsilateral breast cancer, the 5-year and 10-year incidence of local recurrence before PSM were 2.80% and 6.43% for the no-radiotherapy group and 0.97% and 2.96% for the radiotherapy group (*P *< .001); after PSM, the 5-year and 10-year incidence of local recurrence were 2.65% and 6.16% for the no-radiotherapy group and 1.60% and 3.57% for the radiotherapy group (*P < *.001) (Figure S6A, D).

However, if the recurrence was of entirely distant metastasis, the 5-year and 10-year incidence of distant recurrence before PSM were 2.61% and 2.86% for the no-radiotherapy group and 1.27% and 1.68% for the radiotherapy group (*P *< .001); after PSM, the 5-year and 10-year incidence of distant recurrence were 2.42% and 2.70% for the no-radiotherapy group and 1.98% and 2.93% for the radiotherapy group (*P *= .496) (Figure S6B, E).

Before PSM, the 5-year and 10-year incidence of any recurrence were 2.99% and 6.76% for no-radiotherapy and 1.22% and 3.44% for radiotherapy patients (*P *< .001). After PSM, the 5-year incidence of any recurrence were 2.84% and 6.51% for the no-radiotherapy group and 1.93% and 4.56% for the radiotherapy group (*P *< .001) (Figure S6C, F).

## Discussion

Omitting radiotherapy after BCS for breast cancer patients is a controversial topic. Given the prognostic role of molecular phenotypes,^34^ trials like CALGB 9343 and PRIME II have established criteria related to hormone receptor and found that eligible patients could forgo radiotherapy after surgery.^14^^,^^15^ Both the CALGB 9343 and the PRIME II studies found that radiotherapy significantly reduced local recurrence but did not provide survival benefits for patients meeting their criteria.

Unlike those 2 studies, the LUMINA trial focused not on the efficacy of radiotherapy but whether eligible patients’ low recurrence risk made its benefits negligible.^13^ Therefore, criteria they adopted were rigorous, limited to luminal A type, which has the most favorable prognosis among 4 breast cancer subtypes.^35^ Luminal A patients have a 10-year local recurrence incidence around 8%.^36^ Based on these criteria, researchers found 5-year local recurrence incidence of only 2.3% for LUMINA patients. Notably, younger patients were included in their cohort compared with CALGB 9343 and PRIME II cohorts. Another Italian study also explored the recurrence incidence in younger patients. Following up patients aged 55-75 with small tumors for 108 months, it found a recurrence rate of 3.4% for the radiotherapy group and 4.4% for the no-radiotherapy group.^37^

A previous retrospective study reported that among luminal A, clinically low-risk patients treated with tamoxifen, the 10-year local recurrence rate was 5.0% vs 1.3% for the radiotherapy vs no-radiotherapy group (*P *= .42).^35^ However, baseline characteristics between those 2 groups in this study were not well balanced, and the number of patients included was small. This low recurrence rate in the no-radiotherapy group of the study provided justification for the LUMINA trial. Researchers believed that the difference in prognosis caused by radiotherapy and without radiotherapy might be minimal in the LUMINA patients and that establishing a no-radiotherapy group would require a large sample.^13^ From the above 2 studies, it is impossible to know the impact of radiotherapy on the prognosis of LUMINA patients. In addition, patient medication adherence in the clinical trial environment might also differ from the real world.^38^

To address those problems, we established the SEER-LUMINA cohort. The patient composition observed in this real-world cohort differs from that in the LUMINA study. SEER-LUMINA radiotherapy and no-radiotherapy groups showed different patient compositions. Older patients with smaller and well-differentiated tumors seemed more likely to forego radiotherapy. The proportion of patients undergoing SLNB in the no-radiotherapy group was 1.4% higher than that in the radiotherapy group. The NSABP B-32 trial reported a false-negative rate of 9.8% for SLNB.^39^ This suggests that there might be more patients with undetected lymph node metastases in the no-radiotherapy group. However, given the similar proportions of patients undergoing SLNB in both groups, we believe the impact of this possible difference on our results is minimal.

After reducing the imbalance between the 2 groups, we found that no-radiotherapy patients had a higher incidence of local recurrence, poorer OS and BCSS, and slightly lower incidence of contralateral breast cancer, with these differences being statistically significant. The 5-year local recurrence incidence in our research was lower than that in the LUMINA study (0.64% vs 2.3%). However, a prior investigation documented a 10-year local recurrence rate of a mere 1.3% among luminal A patients beyond the age of 60 with T1 stage breast cancers (grade 1 or 2).^35^ According to this precedent, our study’s finding of a less than 1% 5-year local recurrence incidence is quite plausible. We concede the possibility that some instances of recurrence may have gone undiagnosed or undocumented in the SEER database. In light of this, we conducted a sensitivity analysis, which projected an incidence of 2.65%, a figure in alignment with the outcomes of the LUMINA study. Our estimates position the true incidence within the range of 0.64% to 2.65%. Yet, regardless of the precise percentage, the data consistently converge on a singular conclusion: the 5-year cumulative incidence of local recurrence in actual clinical practice is notably lower than 5%. This underscores the broader implication of our findings and their correlation with real-world patient experiences. Furthermore, the recently published PROSPECT study retrospectively analyzed their data based on LUMINA criteria, considering patients aged ≥55 years, grades 1 or 2, non-lobular, PR-positive as potentially satisfying LUMINA criteria due to the absence of Ki-67 records.^16^ The 5-year local recurrence rate for no-radiotherapy patients in the PROSPECT study was 1.0%, and 54% of these patients might meet LUMINA criteria, and patients not fulfilling LUMINA criteria had risks such as grade 3 disease, less than 20% PR positivity, among others.^16^ Hence, the precise 5-year local recurrence in the PROSPECT study for patients potentially eligible for LUMINA criteria might be lower than 1.0%. All these studies reaffirmed that LUMINA patients have a modest risk for local recurrence.

Due to the short follow-up period of the LUMINA study at present, the safety of omitting radiotherapy in those patients has been challenged.^20^^,^^21^ Therefore, we assessed the 10-year outcomes. No-radiotherapy group still had 10-year local and any recurrence incidence below 5%, with the upper boundary of the 95% CI also being below 5%. However, sensitivity analysis showed that the 10-year local and any recurrence incidence were slightly higher than this threshold. Moreover, the ARR for local recurrence at 10 years was 4 times that at 5 years. Therefore, we do believe that a long-time follow-up of 15 years or even more is necessary for the LUMINA trial since it covered younger patients who have longer life expectancy. Nevertheless, the 10-year incidence of SEER-LUMINA patients was still lower than the nearly 10% 10-year local recurrence incidence of the no-radiotherapy group in CALGB 9343 and PRIME II,^14^^,^^19^ once again proving the good prognosis of this group. In addition, since SEER-LUMINA included luminal B patients who were at higher recurrence risk, we speculate that LUMINA patients will have better performance than SEER-LUMINA in terms of 10-year local recurrence.

We then explored risk factors for local recurrence. Beyond radiotherapy, neither tumor size nor grade nor age exerted significant influence on prognoses, echoing findings from PRIME II.^15^ Compared with tumors originating from the upper-outer quadrant, tumors in the lower-outer quadrant exhibited higher recurrence risk with borderline significance. One publication reported better prognoses for upper-outer quadrant tumor patients,^40^ and research from Wu et al. indicated that tumors in the lower inner quadrant were more prone to recurrence.^41^

The contralateral breast cancer incidence for SEER-LUMINA patients was close to LUMINA patients. SEER-LUMINA radiotherapy group displayed a marginally higher rate of contralateral breast cancer compared with the no-radiotherapy cohort, and similar result has been reported in another research.^42^ In PRIME II trial, the 5-year incidence of contralateral breast cancer in the radiotherapy group also exceeded the no-radiotherapy group (1.5% vs 0.7%), albeit without statistical difference.^15^

Previous studies reported that radiotherapy omission did not affect OS in early-stage breast cancer.^15^^,^^37^ However, our analysis showed that the OS and the DFS were markedly higher in the radiotherapy group than in the no-radiotherapy group. Due to the retrospective nature of this study, patients were not randomly assigned to the radiotherapy group or the no-radiotherapy group. Patients with short life expectancy or harboring severe comorbidity might choose not to receive radiotherapy, leading to worse OS and DFS for the no-radiotherapy group as compared to the radiotherapy group, however, this outcome is not caused by radiotherapy itself.

Due to the lack of information on comorbidities, BCSS rather than OS was used to assess impact of radiotherapy on patients’ survival outcomes. We explored patients that might not gain survival benefits from radiotherapy. Patients with tumor size ≤1.4 cm were identified as being at low-risk, and radiotherapy did not result in better BCSS for this population.

Our study is subject to a number of limitations. First, results of LUMINA are generalizable only to women who received endocrine therapy with Ki67 positivity of ≤13.25%, yet these factors were not captured in SEER. Before 2010, however, tamoxifen had been recommended as standard treatment for all ER-positive tumors^43^; hence, we assumed that most SEER-LUMINA patients must have been treated with endocrine therapy. In addition, luminal A type must have accounted for a large proportion of our SEER-LUMINA cohort. Using Ki-67 ≤ 13.25% as the threshold, approximately 59% hormone receptor-positive breast cancer would be categorized as luminal A.^44^ Genomic distinctions between luminal B and luminal A result in a higher recurrence risk for luminal B patients.^35^ Therefore, our study may have overestimated the local recurrence incidence of LUMINA patients. Besides, the SEER database lacks BRCA 1/2 mutation data. A studies showed that the BRCA mutation rate is 1.7% in luminal A and 7.2% in luminal B breast cancer patients.^45^ It is controversial whether BRCA-mutated patients have a higher local recurrence rate after BCS. Some studies indicate no significant difference in local recurrence and overall survival between BRCA-mutated and non-mutated patients after breast-conserving surgery.^46^^,^^47^ Others report that, with longer follow-up, BRCA-mutated patients have a higher local recurrence rate. This may be due to their having a higher probability of new primary tumors, rather than true recurrence. Researchers suggest that radiotherapy in BRCA mutation carriers is at least as effective as in non-carriers.^48^^,^^49^ Additionally, due to the limitations of the database, we were unable to acquire information regarding tumor margins lymphovascular invasion and dose or fractionation of radiotherapy. It is also unclear whether patients had serious comorbidities. Another limitation of our study is the potential absence of recurrence record for some patients who died from breast cancer. This could lead to an underestimation of the recurrence. However, the sensitivity analysis we conducted has partially compensated for this problem.

Breast cancer treatment has entered an era of precision and personalization. Many trials indicate that de-escalation is feasible for low-risk patients. There is no “one-size-fits-all” approach to early ER+ breast cancer; when making treatment plans, patient preferences must be considered.^50^ Despite the encouraging results from those studies, it should be emphasized that treatment de-escalation should only be applied to patients meeting the study-specific eligibility criteria. Besides, other diagnostic and therapeutic measures must be strictly implemented to ensure optimal patient care. For instance, patients exempted from axillary surgery in the SOUND and the INSEMA trials were all planned to undergo whole breast or partial breast radiotherapy.^10^^,^^11^ For LUMINA or LUMINA-like patients, accurate nodal staging should be performed before recommending radiotherapy omission, and the implementation of hormone therapy should be ensured to maintain safety and efficacy. Furthermore, de-de-escalation beyond the current de-escalation requires evidence from rigorously designed prospective studies to ensure patient safety.

## Conclusion

This population-based study elucidated that both the 5-year and the 10-year local recurrence incidence for women aged ≥55 years with early-stage luminal breast cancer who went through BCS without radiotherapy were low. Although radiotherapy significantly reduced recurrence, the absolute reduction was modest. In addition, radiotherapy did not improve BCSS for SEER-LUMINA patients with tumor ≤1.4 cm. However, making treatment decisions is more complicated in the real world and patient preferences must be considered when de-escalating therapies. The results of our study remain to be further validated in a prospective clinical study.

## Supplementary Material

oyaf355_Supplementary_Data

## Data Availability

All data used in this study can be freely accessed from the SEER program (https://seer.cancer.gov/).
