# Supplementary material for: Omitting radiotherapy in LUMINA-like breast cancer after breast conserving surgery: 10-year results of a population-based cohort study
Source: Oncologist. 2025 Oct 22;30(11):oyaf355. doi: 10.1093/oncolo/oyaf355 (PMC12605746; doi:10.1093/oncolo/oyaf355)
Supplement: oyaf355_Supplementary_Data [file oyaf355_supplementary_data.zip › Supplementary Material - Table.doc]

Inclusion and exclusion criteria

Inclusion criteria were: (1) ≥55 years of age (2) breast cancer as the first primary cancer, (3) T1N0M0 stage, (4) ER- and PR-positive and HER2-negative, (5) received BCS for the first diagnosis, (6) grade 1 (well-differentiated) or grade 2 (moderately differentiated) histology, (7) received postoperative radiotherapy or not, (8) no neoadjuvant therapy. Patients with any of following conditions were excluded: (1) lobular carcinoma, (2) ductal carcinoma in situ (DCIS) or micro invasive carcinoma, (3) a second primary breast cancer occurrence within 6 months, (4) less than 1 month of follow-up until death or were lost to follow-up.

Definition and censoring for contralateral breast cancer, BCSS, DFS and OS

Contralateral breast cancer was defined as the occurrence of invasive or DCIS in the contralateral breast. For BCSS, death attributable to breast cancer was considered an event. Individuals who were still alive or who had died from other causes were censored. The SEER database provides cause of death information. DFS events included local or distant recurrences, contralateral breast cancer or deaths from any cause. Patients without these events were censored. For OS, death from any cause was an event. Patients who did not die were censored at the last follow-up date.

Supplemental Table 1 Treatment Outcomes for No-radiotherapy/Radiotherapy Patients before and after PSM

|  | **Local recurrence** | **Contralateral breast cancer** | **Any recurrence** | **OS** | **BCSS** | **DFS** |
| --- | --- | --- | --- | --- | --- | --- |
| 5-year, before PSM, no-radiotherapy | 0.64% (95% CI, 0.44% ~ 0.84%) | 1.67% (95% CI, 1.35% ~ 1.99%) | 0.82% (95% CI, 0.59% ~ 1.04%) | 85.69% (95% CI, 84.85%~ 86.53%) | 98.38% (95% CI, 98.06% ~ 98.69%) | 83.67% (95% CI, 82.79% ~ 84.56%) |
| 5-year, before PSM, radiotherapy | 0.12% (95% CI, 0.07% ~ 0.17%) | 1.64% (95% CI, 1.47% ~ 1.81%) | 0.35% (95% CI, 0.27% ~ 0.43%) | 96.27% (95% CI, 96.01% ~ 96.53%) | 99.42% (95% CI, 99.31% ~ 99.52%) | 94.62% (95% CI, 94.32% ~ 94.93) |
| 5-year, after PSM, no-radiotherapy | 0.65% (95% CI, 0.44% ~ 0.87%) | 1.77% (95% CI, 1.42%~ 2.12%) | 0.83% (95% CI, 0.59% ~ 1.07%) | 87.48% (95% CI, 86.64% ~ 88.33%) | 98.51% (95% CI, 98.19% ~ 98.83%) | 85.36% (95% CI, 84.46% ~ 86.27%) |
| 5-year, after PSM, radiotherapy | 0.14% (95% CI, 0.04% ~ 0.24%) | 2.19% (95% CI, 1.81%~ 2.57%) | 0.46% (95% CI, 0.28% ~ 0.63%) | 93.60% (95% CI, 92.98% ~ 94.23%) | 99.05% (95% CI, 98.80% ~ 99.31%) | 91.47% (95% CI, 90.76% ~ 92.18%) |
| 10-year, before PSM, no-radiotherapy | 3.75% (95% CI, 2.94% ~ 4.55%) | 3.23% (95% CI, 2.64% ~ 3.81%) | 4.08% (95% CI, 3.25% ~ 4.90%) | 63.59% (95% CI, 61.79% ~ 65.45%) | 95.76% (95% CI, 94.93% ~ 96.59%) | 59.16% (95% CI, 57.32% ~ 61.06%) |
| 10-year, before PSM, radiotherapy | 1.68% (95% CI, 1.40% ~ 1.96%) | 4.02% (95% CI, 3.62% ~ 4.41%) | 2.17% (95% CI, 1.86% ~ 2.48%) | 86.14% (95% CI, 85.42% ~ 86.87%) | 97.97% (95% CI, 97.67% ~ 98.27%) | 81.43% (95% CI, 80.63% ~ 82.25%) |
| 10-year, after PSM, no-radiotherapy | 3.68% (95% CI, 2.87% ~ 4.49%) | 3.34% (95% CI, 2.72% ~ 3.97%) | 4.03% (95% CI, 3.19% ~ 4.87%) | 66.73% (95% CI, 64.85% ~ 68.66%) | 96.11% (95% CI, 95.28% ~ 96.95%) | 62.18% (95% CI, 60.26% ~ 64.16%) |
| 10-year, after PSM, radiotherapy | 1.54% (95% CI, 1.00% ~ 2.07%) | 5.25% (95% CI, 4.29% ~ 6.21%) | 2.50% (95% CI, 1.83% ~ 3.17%) | 75.57% (95% CI, 73.79% ~ 77.38%) | 96.34% (95% CI, 95.51% ~ 97.19%) | 70.76% (95% CI, 68.89% ~ 72.68%) |

Supplemental Table 2. Characteristics of the Patients after PSM

| **Variable** | **Received radiotherapy after breast conserving surgery?** | | ***P*** |
| --- | --- | --- | --- |
|  | **No (n =5954)** | **Yes (n =5954)** |  |
| Age, n (%) |  |  | 0.972 |
| 55 to <60 years | 534 (9.0) | 526 (8.8) |  |
| 60 to <65 years | 682 (11.5) | 681 (11.4) |  |
| 65 to <70 years | 908 (15.3) | 897 (15.1) |  |
| 70 to <75 years | 1326 (22.3) | 1309 (22.0) |  |
| 75 to <80 years | 1233 (20.7) | 1273 (21.4) |  |
| ≥80 years | 1271 (21.3) | 1268 (21.3) |  |
| Year of diagnosis, n (%) |  |  | 0.485 |
| 2010 | 824 (13.8) | 818 (13.7) |  |
| 2011 | 926 (15.6) | 942 (15.8) |  |
| 2012 | 954 (16.0) | 956 (16.1) |  |
| 2013 | 986 (16.6) | 1046 (17.6) |  |
| 2014 | 1120 (18.8) | 1046 (17.6) |  |
| 2015 | 1144 (19.2) | 1146 (19.2) |  |
| Chemotherapy, n (%) |  |  | 1.000 |
| No/Unknown | 5827 (97.9) | 5826 (97.9) |  |
| Yes | 127 (2.1) | 128 (2.1) |  |
| Tumor size, n (%) |  |  | 0.240 |
| ≤0.5 cm | 909 (15.3) | 860 (14.4) |  |
| 0.5–1.0 cm | 2329 (39.1) | 2295 (38.5) |  |
| 1.1–2.0 cm | 2716 (45.6) | 2799 (47.0) |  |
| Tumor grade, n (%) |  |  | 0.180 |
| Well differentiated; Grade 1 | 3194 (53.6) | 3120 (52.4) |  |
| Moderately differentiated; Grade 2 | 2760 (46.4) | 2834 (47.6) |  |
| Histologic cancer type, n (%) |  |  | 0.140 |
| Ductal | 5335 (89.6) | 5407 (90.8) |  |
| Tubular | 106 (1.8) | 90 (1.5) |  |
| Mucinous | 350 (5.9) | 321 (5.4) |  |
| Other | 163 (2.7) | 136 (2.3) |  |
| Primary Site, n (%) |  |  | 0.014 |
| Upper-outer | 2047 (34.4) | 2068 (34.7) |  |
| Upper-inner | 984 (16.5) | 940 (15.8) |  |
| Lower-outer | 461 (7.7) | 409 (6.9) |  |
| Lower-inner | 432 (7.3) | 419 (7.0) |  |
| Central portion & Nipple | 205 (3.4) | 161 (2.7) |  |
| others | 1825 (30.7) | 1957 (32.9) |  |
| Laterality, n (%) |  |  | 0.769 |
| Left | 2991 (50.2) | 3008 (50.5) |  |
| Right | 2963 (49.8) | 2946 (49.5) |  |
| Race, n (%) |  |  | 0.047 |
| White | 5042 (84.7) | 5149 (86.5) |  |
| Black | 387 (6.5) | 338 (5.7) |  |
| Asian or Pacific Islander | 455 (7.6) | 408 (6.9) |  |
| Unknown/others | 70 (1.2) | 59 (1.0) |  |

Supplemental Table 3. Univariate and Multivariate Analyses of the Local Recurrence for SEER-LUMINA Patients after PSM

| **Variable** | | **n** | **HR (univariable)** | **HR (multivariable)** |
| --- | --- | --- | --- | --- |
| Age | <65 years | 2423 (20.3%) | Reference |  |
|  | ≥65 years | 9485 (79.7%) | 1.11 (0.76-1.62, *P*=0.589) |  |
| Year of diagnosis | 2010-2012 | 5420 (45.5%) | Reference |  |
|  | 2013-2015 | 6488 (54.5%) | 0.78 (0.55-1.10, *P*=0.156) |  |
| Chemotherapy | No/Unknown | 11653 (97.9%) | Reference |  |
|  | Yes | 255 (2.1%) | 0.24 (0.03-1.71, *P*=0.154) |  |
| Tumor size | ≤1.0 cm | 6393 (53.7%) | Reference |  |
|  | >1.0 cm | 5515 (46.3%) | 0.85 (0.62-1.16, *P*=0.313) |  |
| Tumor grade | Grade 1 | 6314 (53.0%) | Reference |  |
|  | Grade 2 | 5594 (47.0%) | 0.95 (0.69-1.29, *P*=0.728) |  |
| Histologic cancer type | Ductal | 10742 (90.2%) | Reference |  |
|  | Mucinous | 671 (5.6%) | 0.95 (0.47-1.94, *P*=0.895) |  |
|  | Other | 299 (2.5%) | 1.55 (0.69-3.51, *P*=0.292) |  |
|  | Tubular | 196 (1.6%) | 1.11 (0.36-3.50, *P*=0.852) |  |
| Primary Site | Upper-outer | 4115 (34.6%) | Reference | Reference |
|  | Central portion & Nipple | 366 (3.1%) | 1.11 (0.44-2.78, *P*=0.824) | 1.05 (0.42-2.63, *P*=0.919) |
|  | Lower-inner | 851 (7.1%) | 1.24 (0.67-2.28, *P*=0.492) | 1.24 (0.67-2.28, *P*=0.496) |
|  | Lower-outer | 870 (7.3%) | 1.70 (0.99-2.91, *P*=0.054) | 1.70 (0.99-2.92, *P*=0.053) |
|  | Upper-inner | 1924 (16.2%) | 1.06 (0.66-1.71, *P*=0.795) | 1.07 (0.66-1.71, *P*=0.790) |
|  | others | 3782 (31.8%) | 1.06 (0.71-1.57, *P*=0.779) | 1.08 (0.73-1.60, *P*=0.700) |
| Laterality | Left | 6002 (50.4%) | Reference | Reference |
|  | Right | 5916 (49.6%) | 1.31 (0.96-1.78, *P*=0.092) | 1.31 (0.96-1.78, *P*=0.092) |
| Race | White | 10191 (85.6%) | Reference |  |
|  | Asian or Pacific Islander | 863 (7.2%) | 0.71 (0.36-1.39, *P*=0.319) |  |
|  | Black | 725 (6.1%) | 0.81 (0.40-1.65, *P*=0.563) |  |
|  | Unknown/others | 129 (1.1%) | 0.51 (0.07-3.68, *P*=0.508) |  |
| Radiotherapy | No | 5954 (50.0%) | Reference | Reference |
|  | Yes | 5954 (50.0%) | 0.35 (0.25-0.49, *P* <0.001) | 0.35 (0.25-0.49, *P* <0.001) |

Supplemental Table 4. Characteristics of the Patients with Tumor Size ≤1.4 cm after PSM

| **Variable** | **Received radiotherapy after breast conserving surgery?** | | ***P*** |
| --- | --- | --- | --- |
|  | **No (n =4580)** | **Yes (n =4580)** |  |
| Age, n (%) |  |  | 0.881 |
| 55 to <60 years | 375 ( 8.2) | 355 ( 7.8) |  |
| 60 to <65 years | 534 (11.7) | 545 (11.9) |  |
| 65 to <70 years | 696 (15.2) | 703 (15.3) |  |
| 70 to <75 years | 1080 (23.6) | 1047 (22.9) |  |
| 75 to <80 years | 972 (21.2) | 1005 (21.9) |  |
| ≥80 years | 923 (20.2) | 925 (20.2) |  |
| Year of diagnosis, n (%) |  |  | 0.040 |
| 2010 | 615 (13.4) | 629 (13.7) |  |
| 2011 | 662 (14.5) | 753 (16.4) |  |
| 2012 | 801 (17.5) | 711 (15.5) |  |
| 2013 | 764 (16.7) | 770 (16.8) |  |
| 2014 | 837 (18.3) | 816 (17.8) |  |
| 2015 | 901 (19.7) | 901 (19.7) |  |
| Chemotherapy, n (%) |  |  | 0.437 |
| No/Unknown | 4517 (98.6) | 4507 (98.4) |  |
| Yes | 63 ( 1.4) | 73 ( 1.6) |  |
| Tumor size, n (%) |  |  | 0.753 |
| ≤0.5 cm | 898 (19.6) | 883 (19.3) |  |
| 0.5–1.0 cm | 2323 (50.7) | 2359 (51.5) |  |
| 1.1–2.0 cm | 1359 (29.7) | 1338 (29.2) |  |
| Tumor grade, n (%) |  |  | 0.899 |
| Well differentiated; Grade 1 | 2620 (57.2) | 2627 (57.4) |  |
| Moderately differentiated; Grade 2 | 1960 (42.8) | 1953 (42.6) |  |
| Histologic cancer type, n (%) |  |  | 0.158 |
| Ductal | 4126 (90.1) | 4186 (91.4) |  |
| Tubular | 98 ( 2.1) | 80 ( 1.7) |  |
| Mucinous | 240 ( 5.2) | 218 ( 4.8) |  |
| Other | 116 ( 2.5) | 96 ( 2.1) |  |
| Primary Site, n (%) |  |  | 0.242 |
| Upper-outer | 1646 (35.9) | 1615 (35.3) |  |
| Upper-inner | 701 (15.3) | 699 (15.3) |  |
| Lower-outer | 348 ( 7.6) | 312 ( 6.8) |  |
| Lower-inner | 320 ( 7.0) | 332 ( 7.2) |  |
| Central portion & Nipple | 158 ( 3.4) | 134 ( 2.9) |  |
| others | 1407 (30.7) | 1488 (32.5) |  |
| Laterality, n (%) |  |  | 0.095 |
| Left | 2287 (49.9) | 2368 (51.7) |  |
| Right | 2293 (50.1) | 2212 (48.3) |  |
| Race, n (%) |  |  | 0.097 |
| White | 3894 (85.0) | 3977 (86.8) |  |
| Black | 303 ( 6.6) | 261 ( 5.7) |  |
| Asian or Pacific Islander | 332 ( 7.2) | 296 ( 6.5) |  |
| Unknown/others | 51 ( 1.1) | 46 ( 1.0) |  |
