# Supplementary material for: Omitting radiotherapy in LUMINA-like breast cancer after breast conserving surgery: 10-year results of a population-based cohort study
Source: Oncologist. 2025 Oct 22;30(11):oyaf355. doi: 10.1093/oncolo/oyaf355 (PMC12605746; doi:10.1093/oncolo/oyaf355)
Supplement: oyaf355_Supplementary_Data [file oyaf355_supplementary_data.zip › Supplementary Material - Figure.doc]

Supplemental Figure 1. Risk of Local Recurrence in No-radiotherapy Patients. The cumulative risk of local recurrence in the SEER cohort is stratified by (A) year of diagnosis, (B) chemotherapy, (C) histologic cancer type, (D) primary site, (E) laterality, (F) race

Supplemental Figure 2. Risk of Local Recurrence in Radiotherapy Patients. The cumulative risk of local recurrence in the SEER cohort is stratified by (A) year of diagnosis, (B) chemotherapy, (C) histologic cancer type, (D) primary site, (E) laterality, (F) race

Supplemental Figure 3. Cumulative Incidence of Non−breast−cancer−related Death after PSM

Supplemental Figure 4. X-tile analysis for tumor size stratification: (A) The optimal cutoff value, (B) numbers of patients in low-risk and high-risk subgroups.

Supplemental Figure 5. BCSS for patients with tumors ≤1.4 cm after PSM

Supplemental Figure 6. Sensitivity Analysis for Recurrence.
